# **Effects of live-performed sansula music versus storytelling on physiological and behavioral parameters in preterm infants: a randomized controlled trial**

**DOI:** 10.1038/s41598-025-25875-8

**Published:** 2025-11-07

**Authors:** Alisa Weinkoetz, Nina Jacqueline Klasen, Ursula Felderhoff-Mueser, Nora Bruns, Susann Kobus

**Affiliations:** 1 Department of Pediatrics I, University Hospital, University of Duisburg-Essen, Essen, Germany; 2 Faculty of Medicine, Center for Translational Neuro- and Behavioral Sciences, C-TNBS, University of Duisburg-Essen, Essen, Germany; 3 Center of Artistic Therapy, University Medicine Essen, Essen, Germany

**Keywords:** Music therapy, Sansula, Preterm infants, Neonatal intensive care unit, Storytelling, Neonatology, Diseases, Health care, Medical research

## Abstract

Evidence supporting the benefits of music therapy for preterm infants during neonatal care is growing. This study aimed to compare the effects of live-performed sansula music (SM) to storytelling (ST) as an alternative caregiving intervention on physiological and behavioral responses. The study was conducted as a randomized controlled trial (RCT), enrolling preterm infants born from 32 + 0 to 36 + 6 weeks of gestation. Infants were block-randomized to either receive SM or ST. Both interventions were performed live and provided three to four times per week on average. Vital signs and behavioral state (assessed using the COMFORTneo scale) were documented before, during, and after each intervention. Sixty preterm infants were enrolled in the study: 30 received SM and 30 received ST. We observed positive effects on vital signs and behavioral state in both groups, although the effects were importantly less pronounced in the ST group compared to the SM group. Heart rate decreased during both SM and ST interventions (mean difference (MD) (baseline–after): SM -9.90 bpm (95% CI -11.14 to -8.66); ST -2.85 bpm (-3.79 to -1.92)). Respiratory rate also decreased for both groups (MD: SM -8.60 breaths/min (-9.60 to -7.60); ST -2.49 breaths/min (-3.30 to -1.68)). Oxygen saturation increased (MD: SM +1.01% (0.82 to 1.20); ST +0.25% (0.11 to 0.39)). The COMFORTneo score decreased (MD: SM -5.40 (-5.95 to -4.86); ST -2.26 (-2.74 to -1.77)). Both, live-performed sansula music as a therapeutic intervention and storytelling, positively impacted the immediate well-being of preterm infants. However, the benefits of live-performed sansula music on physiological and behavioral parameters compared to storytelling as a caregiving alternative were significantly higher.

**Supplementary Information:**

The online version contains supplementary material available at 10.1038/s41598-025-25875-8.

## Introduction

 Worldwide, an estimated 13.4 million newborns were born preterm in 2020, which corresponds to one in ten infants^1^. According to the World Health Organization (WHO), a preterm infant is born before the completion of 37 weeks of gestation^2^. Complications due to preterm birth remain the leading cause of mortality among children under the age of five globally^3^.

From the moment of birth, preterm infants require special care to support their immature organ systems as they adapt to extrauterine life. They are highly sensitive to external stimuli, such as light and noise^4^. Many of them require advanced care in the neonatal intensive care unit (NICU), where they are exposed to unnatural noises and sound levels that exceed the recommended thresholds, leading to sensory overstimulation^5^. Combined with prolonged separation from parents and painful or stressful procedures, the NICU environment contributes to neonatal stress, which has been linked to adverse emotional, cognitive, motor, and structural brain development^6^.

Compared to full-term infants, preterm infants are at a higher risk for various medical conditions, including cardiovascular and respiratory diseases^7^, neurological and psychiatric disorders^8,9^, and visual and auditory impairments^10^. These conditions, in turn, increase the likelihood of impaired long-term health, education, and social outcomes^11^.

Thus, it is essential to identify interventions that effectively reduce stress in preterm infants in order to optimize neonatal clinical care and improve short- and long-term outcomes.

Family support and care, as well as complementary interventions like kangaroo care, have improved outcomes, including immediate well-being as well as survival rates^12–14^. Likewise, music therapy has gained increasing recognition in recent years as a non-invasive adjunctive intervention. Research indicates that music-based interventions have the potential to reduce stress and pain in newborns, infants, and children, with growing evidence supporting their beneficial effects on preterm infants^15,16^.

To date, most studies involving preterm infants have compared music-based interventions to standard care. A meta-analysis by Yue et al. (2021)^16^ found significant positive effects on heart rate, respiratory rate, stress level, oral feeding volume, and maternal anxiety. Costa et al. (2022)^17^ showed beneficial effects on cardiac and respiratory function, sleep patterns, weight gain, feeding behaviors, pain, and brain activity in a systematic review .

It remains unclear whether these benefits result from the intervention itself or from the broader concept of providing individualized care and attention. This study aimed to compare the effects of live-performed sansula music (SM) as a therapeutic intervention versus storytelling (ST), as caregiving alternative, on preterm infants with respect to changes in vital signs and behavioral state.

## Materials and methods

### Study design

The study was performed as a prospective, randomized, controlled clinical trial. Sixty infants were recruited and randomized in a 1:1 ratio to receive either standard care plus sansula music (SM) or standard care plus storytelling (ST).

### Sample size

A sample size of 27 participants per group was calculated using a power analysis which relied on the study’s primary outcomes (vital signs and COMFORTneo score before versus after SM and ST). As input parameters, an effect size (Cohen’s d) of 0.6, a significance level (α) of 0.05, and a statistical power of 70% were assumed based on previous experience^18^. In total, 30 participants were enrolled per group.

### Eligibility and recruitment

The trial was conducted in the NICU and the neonatal wards of the University Hospital Essen, Germany. Preterm infants born at the University Hospital Essen between April 2023 and January 2024, with a gestational age (GA) of 32 + 0 to 36 + 6 weeks, were eligible to enroll in this study, provided that a hospital stay of at least seven days was expected.

To ensure a balanced allocation of participants to the two intervention groups, sansula music (SM) and storytelling (ST), block randomization was employed. This method avoids groups of different sizes, thereby enhancing the comparability of the two groups. Block randomization with 20 participants per block was performed. For every block, 20 consecutively numbered envelopes were prepared, with the numbers corresponding to each infant’s study number. Ten sheets for SM and ten for ST were randomly shuffled and placed into the numbered envelopes, one sheet per envelope. This procedure was repeated three times, resulting in a total of 60 envelopes for 60 infants. Upon enrollment, each infant was assigned a consecutive study number. The envelope matching the infant’s study number was then opened, revealing the assigned intervention.

Before being enrolled in the trial, written informed consent was obtained from at least one parent of each participant. The study was approved by the local Ethics Committee of the Medical Faculty of the University of Duisburg-Essen (23-11159-BO) at 23/04/2023 and registered in the German Clinical Trials Registry DRKS00036274 (25/02/2025). The CONSORT guidelines were applied. The study was conducted in accordance with the Declaration of Helsinki.

### Live-performed sansula music as a therapeutic intervention

The sansula music (SM) group received live-performed music therapy using instrumental sansula music an average of three to four times per week, with each session lasting about 25 minutes on average. We aimed to start the therapy sessions within the first ten days after birth, and therapy was continued until the infant’s discharge or transfer to another ward. Sansula music therapy sessions were performed by the music therapist and two trained medical students as one-on-one sessions for each infant. Infants were not assigned to a single music provider. Instead, each session was delivered by whichever provider was available at that time. In the case of twins or triplets lying side by side in one bed and assigned to the same intervention group, the intervention was carried out simultaneously by maintaining the same distance from each infant and alternately directing the instrument toward one infant and then the other. The instrument used for the live-performed sessions was the Sansula, a modified version of the Kalimba, also known as thumb piano. It consists of a wooden soundboard with attached metal tines. Unlike the traditional Kalimba, the Sansula is mounted on a drumhead, enhancing its resonance. It is held in both hands and played by plucking the metal tines with the thumbs. The generated sound is gentle and sustained, with a resonant, soft timbre. The Sansula used in this study was tuned in A minor, and of its nine notes, the three lowest, A, C’, and E’, were played predominantly. The music was improvised without pre-specified melodies, allowing the infant’s reactions to guide the music. During the therapy session, the infant remained either in the incubator or a (heated) cot or was held by a parent or relative. The therapy provider performed the session while either standing or sitting beside the infant. The instrument was held approximately 20–30 cm from the infant, directed toward the infant’s head. This ensured that the infant was surrounded by both sound and vibration.

### Storytelling intervention

Storytelling (ST) was the alternative live-performed intervention. Similar to the SM intervention, the first session of storytelling was scheduled shortly after the infant’s birth and was provided on average three to four times per week. The therapist or medical student stood or sat beside the infant, and in cases involving multiples lying in the same bed and assigned to the same intervention group, the ST sessions were conducted together. The three stories “Träum schön, kleiner Bär” [Sweet dreams, little bear], “Wovon kleine Schwalben träumen” [What little swallows dream of], and “Der Igel Isidor” [Isidor the hedgehog] from the German book Kuschelgeschichten zur guten Nacht [Bedtime cuddle stories] (Cratzius, 2017)^19^, were read to the infant in a session lasting approximately 25 min on average. The stories were read at a slow pace, with improvised additions made during and after reading. They were narrated in a soft, pleasant voice, adjusted to the infant’s response.

### Vital signs

As physiological outcome parameters, heart rate, respiratory rate, and oxygen saturation were assessed. As part of routine care, these vital signs were continuously monitored. For this study, the mean values of one-minute intervals displayed on the infants’ patient monitors were documented, starting ten minutes before the intervention, throughout the intervention, and until ten minutes after. For each session, mean values for the three time periods before, during, and after the intervention were calculated and used for further analysis.

### COMFORTneo scale

The COMFORTneo scale is a clinical observation tool for evaluating pain and distress in neonatal infants^20^. It comprises seven elements: alertness, calmness/agitation, respiratory response, crying, body movement, facial tension, and (body) muscle tone. The item crying applies only to infants who breathe spontaneously, while the respiratory response is assessed in mechanically ventilated infants. Therefore, six out of seven items are used. Each category is scored from one to five, resulting in a total score from 6 to 30. A score of 14 or higher indicates the presence of pain or distress^20^. In this study, the preterm infant’s behavioral state was assessed using the COMFORTneo scale directly before, during, and shortly after the SM and ST interventions.

Furthermore, we documented whether the infants had physical contact in form of being touched or held and we recorded their state of wakefulness (asleep/awake) at each of the three time points (before/during/after).

### Statistical analysis

Continuous variables are presented as mean with standard deviation (SD) or confidence intervals (CI) if normally distributed and as median with interquartile range (IQR) and range if skewed. To estimate the effect of live-performed sansula music compared to storytelling, we calculated the adjusted mean differences of baseline versus post-therapy values for both intervention groups. These values were first calculated individually for each patient and subsequently averaged. A multivariable linear mixed model was used with fixed effects for physical contact during the session and wakefulness before the session. We accounted for repeated measurements within one individual by adding the study number as a random effect, as previously applied^21^. Statistical analyses and figure generation were performed using SAS Enterprise Guide 8.4 (SAS Institute Inc., Cary, NC, United States).

## Results

### Patients

**Fig. 1:**
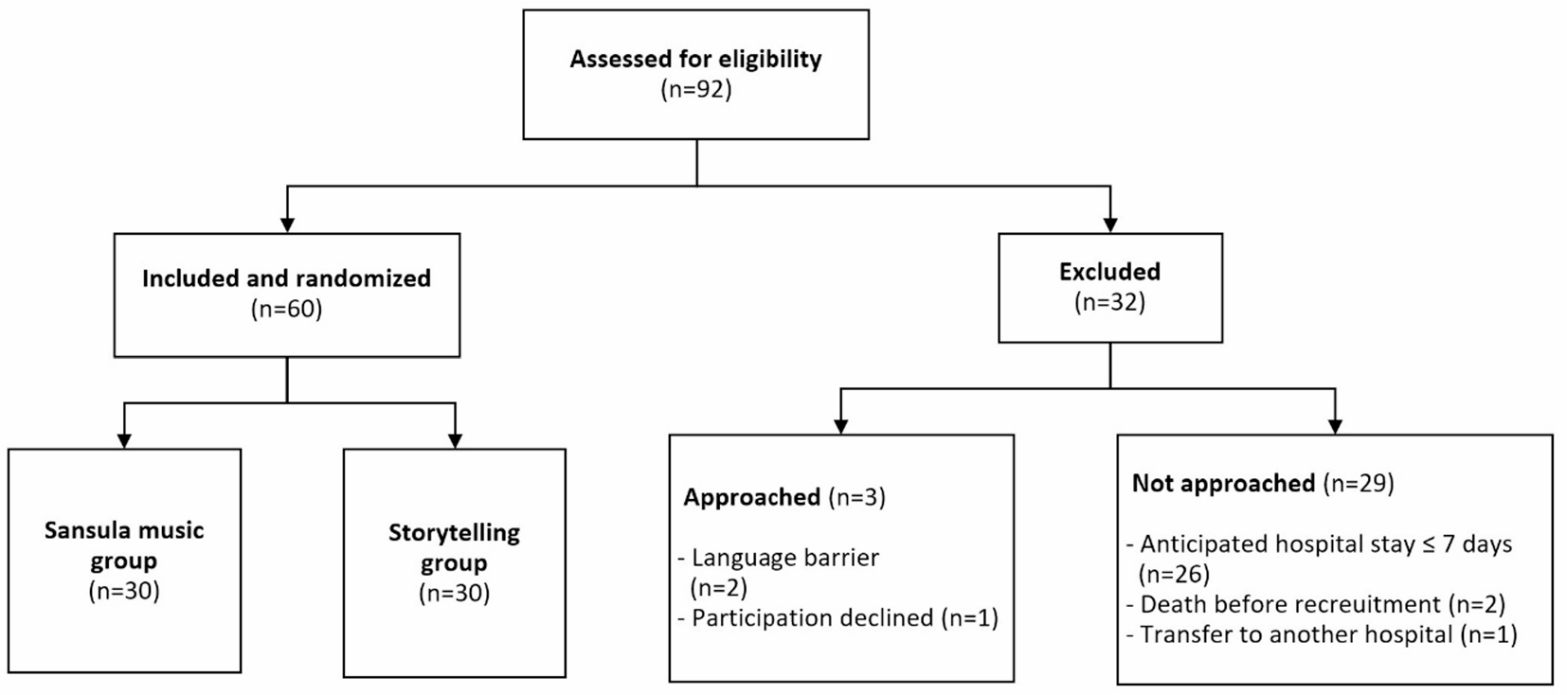
Flowchart of the included and excluded patients.

Between April 2023 and January 2024, 92 infants with a gestational age ranging from 32 + 0 to 36 + 6 weeks were born at the University Hospital Essen and potentially eligible for inclusion in our study. Thirty-two of these infants were excluded from the study. The primary reason for exclusion was an expectedly short hospital stay ≤ 7 days (*n* = 26), followed by death before recruitment (*n* = 2), the inability to obtain parental consent due to a language barrier (*n* = 2), parental refusal to participate (*n* = 1), and transfer to another hospital (*n* = 1). Sixty infants were enrolled in the study and randomly assigned in a 1:1 ratio to one of two study arms, with 30 infants in the SM group and 30 infants in the ST group. Study enrollment and randomization are depicted in Fig. 1 as a flowchart.

The mean (SD) gestational age at birth was similar across all groups: 34.6 (± 1.1) weeks in the SM group, 34.3 (± 1.2) weeks in the ST group, and 34.7 (± 1.4) weeks in the excluded group. Similarly, no major differences were found in birth weight between the groups: 2231 (± 579) g in the SM group, 2149 (± 439) g in the ST group, and 2321 (± 402) g in the excluded group. However, differences were observed in sex distribution: 27% male and 73% female in the SM group, and 57% male and 43% female in the ST group. Other clinical parameters, such as the APGAR score and the umbilical arterial pH, showed similar values across all three groups. Overall, the group characteristics were largely balanced. Detailed clinical characteristics of both the included and excluded patients, categorized by their respective groups, are presented in Table 1.

**Table 1:** Clinical characteristics of the included and excluded patients.

	Sansula music group (*n* = 30)	Storytelling group (*n* = 30)	Excluded patients (*n* = 32)
Male, n (%)	8 (27)	17 (57)	14 (44)
Female, n (%)	22 (73)	13 (43)	18 (56)
GA at birth, weeks, mean ± SD (range)	34.6 ± 1.1 (32 + 3–36 + 4)	34.3 ± 1.2 (32 + 0–36 + 5)	34.7 ± 1.4 (32 + 0–36 + 6)
Birth weight, g, mean ± SD (range)	2231 ± 579 (1300–4005)	2149 ± 439 (1230–3300)	2321 ± 402 (1480–3050)
Body length at birth, cm, mean ± SD (range)	45.4 ± 2.6 (33.0–52.0)	44.8 ± 3.0 (37.0–51.0)	45.4 ± 2.9 (39.0–50.0)
Head circumference at birth, cm, mean ± SD (range)	31.8 ± 1.7 (28.0–35.0)	31.6 ± 2.1 (28.0–35.5)	31.9 ± 1.6 (29.0–35.0)
APGAR score at 1 min., median (range)	8.0 (2.0–10.0)	7.0 (2.0–10.0)	7.0 (0.0–10.0)
APGAR score at 5 min., median (range)	8.5 (3.0–10.0)	8.0 (5.0–10.0)	8.0 (4.0–10.0)
APGAR score at 10 min., median (range)	9.5 (6.0–10.0)	9.0 (7.0–10.0)	9.0 (4.0–10.0)
Cesarean section, n (%)	28 (93)	30 (100)	29 (91)
Umbilical artery pH, mean ± SD (range)	7.3 ± 0.1 (7.0–7.4)	7.3 ± 0.1 (7.1–7.4)	7.3 ± 0.1 (6.8–7.4)
Weight at discharge, g, mean ± SD (range)	2541 ± 450 (1885–3720)	2513 ± 340 (2050–3715)	2399 ± 271 (1755–3025)
Body length at discharge, cm, mean ± SD (range)	48.3 ± 2.6 (43.5–55.0)	47.0 ± 3.6 (32.5–53.0)	46.6 ± 2.4 (41.0–51.0)
Head circumference at discharge, cm, mean ± SD (range)	33.1 ± 1.7 (29.5–37.0)	33.5 ± 3.0 (31.0–48.0)	32.5 ± 1.5 (29.0–35.0)

GA = gestational age. Data are presented as mean and standard deviation, if not indicated otherwise.

### Interventions

A total of 552 sessions were conducted, with 303 sessions performed as live sansula music (SM) and 249 sessions as storytelling (ST). SM sessions lasted a median of 24 min (IQR 22–26; range 20–35), and ST sessions 25 min (IQR 19–26; range 11–29). The infants received an average of three to four interventions per week. The first session was administered on median day 4.5 of life for SM (IQR 2–8; range 1–26) and on median day 3 for ST (IQR 3–4; range 2–16), the interventions were continued until the infants’ discharge or transfer to another ward. Because the length of hospital stay from birth to discharge varied among the infants, the total number of interventions differed between the groups. Infants in the SM group received in total a median of 9.5 SM sessions (IQR 7–12.75; range 1–29), and those in the ST group a median of 7 sessions (IQR 6–9; range 3–32).

### Vital sign response

**Fig. 2:**
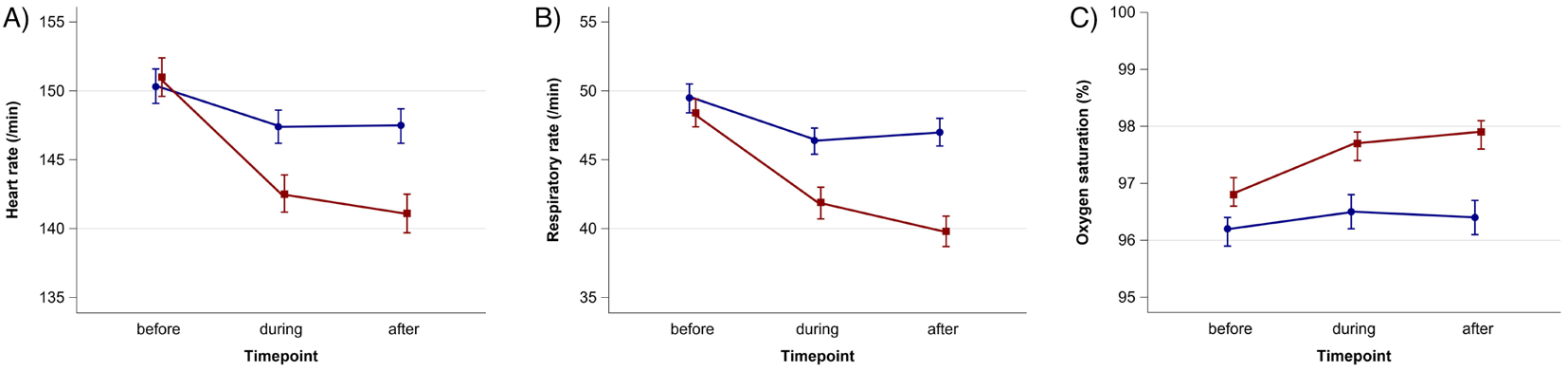
Heart rate (**A**), respiratory rate (**B**), and oxygen saturation (**C**) before, during, and after sansula music sessions (red) and storytelling sessions (blue).

Heart rate and respiratory rate declined during both SM and ST interventions, while oxygen saturation increased. These changes lasted beyond the therapy session and were more pronounced in the SM group, which exhibited an additional post-therapy reduction in heart rate and respiratory rate, and a further increase in oxygen saturation. The observed effects were stronger in SM than in ST (Fig. 2; Table 2).

The mean difference (pre- vs. post-therapy) and the relative change from baseline were larger for SM compared to ST for all vital signs (Table 2). After adjustment, the mean difference in the SM group compared to the ST group was 7.3 beats per minute (95% CI 5.2–9.4) greater for the heart rate, 6.3 breaths per minute (4.4–8.1) greater for the respiratory rate, and 0.9% (0.5–1.2) larger for the oxygen saturation.

**Table 2:** Heart rate, respiratory rate, oxygen saturation, and comfortneo score before (baseline), during, and after Sansula music sessions and storytelling sessions.

Vital sign	Therapy	Sessions (*n*)	Baseline mean (95% CI)	During session mean (95% CI)	After session mean (95% CI)	Mean difference (baseline–after) (95% CI)	Relative change from baseline (%)
Heart rate (beats/ min)	Sansula music	303	151.01 (149.57–152.45)	142.55 (141.21–143.89)	141.11 (139.69–142.53)	-9.90 (-11.14–(-8.66))	-6.33
	Storytelling	249	150.33 (149.09–151.57)	147.39 (146.21–148.56)	147.47 (146.21–148.73)	-2.85 (-3.79–(-1.92))	-1.79
Respiratory rate (breaths/ min)	Sansula music	303	48.44 (47.44–49.43)	41.85 (40.73–42.98)	39.84 (38.74–40.94)	-8.60 (-9.60–(-7.60))	-16.98
	Storytelling	249	49.47 (48.42–50.52)	46.37 (45.42–47.32)	46.98 (45.98–47.98)	-2.49 (-3.30–(-1.68))	-4.16
SpO_2_ (%)	Sansula music	303	96.84 (96.61–97.07)	97.68 (97.45–97.91)	97.85 (97.61–98.08)	+1.01 (0.82–1.20)	+1.06
	Storytelling	249	96.17 (95.90–96.45)	96.48 (96.20–96.75)	96.42 (96.14–96.70)	+0.25 (0.11–0.39)	+0.26
COMFORTneo score	Sansula music	303	12.71 (12.20–13.21)	8.73 (8.42–9.03)	7.30 (7.04–7.56)	-5.40 (-5.95–(-4.86))	-34.93
	Storytelling	249	12.90 (12.37–13.43)	10.68 (10.28–11.07)	10.64 (10.20–11.08)	-2.26 (-2.74–(-1.77))	-12.33

CI = confidence interval. SpO2 = oxygen saturation.

### Behavioral response

**Fig. 3:**
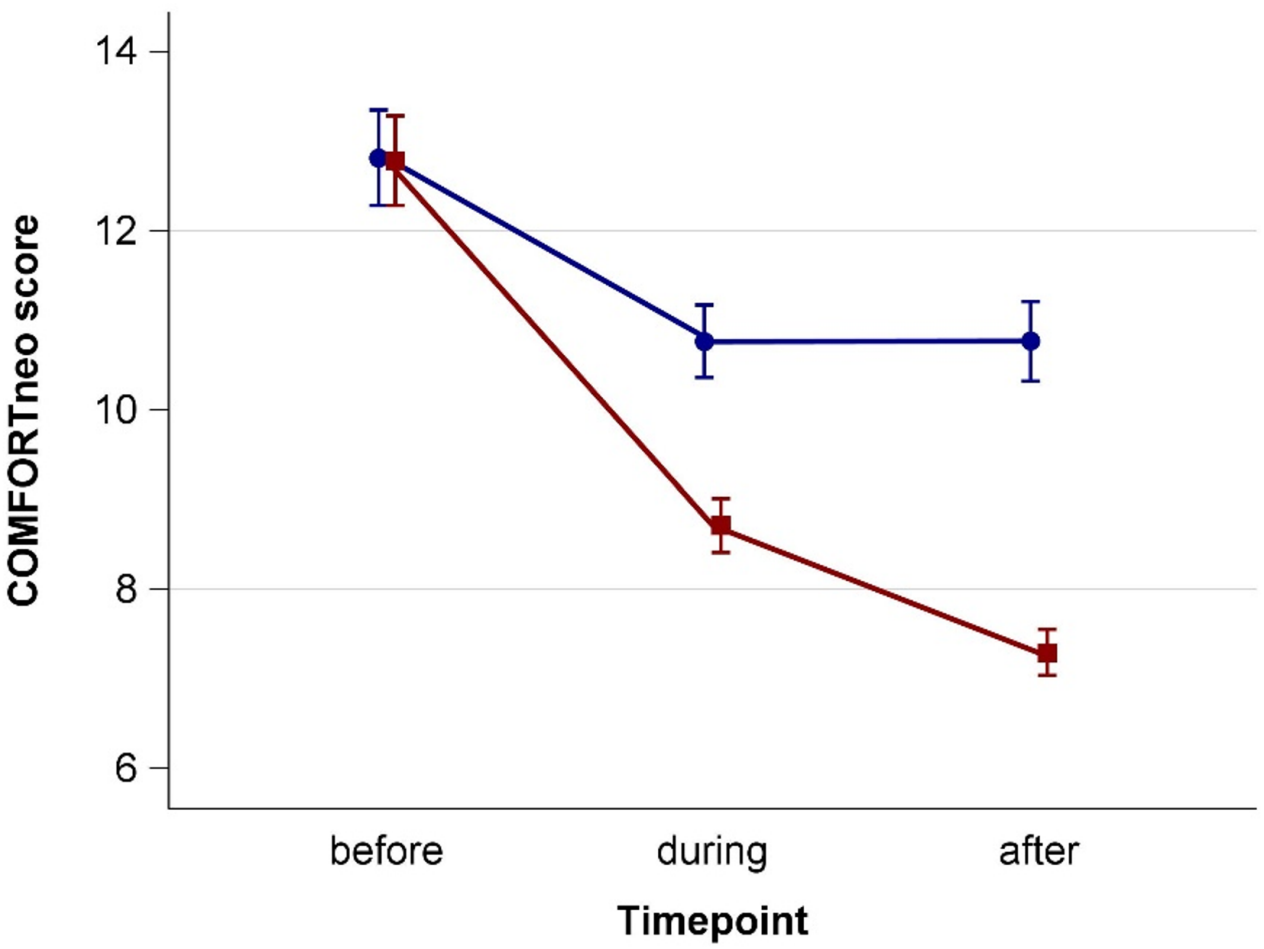
COMFORTneo score before, during, and after sansula music sessions (red) and storytelling sessions (blue).

Both, SM and ST group, showed similar baseline COMFORTneo scores, which decreased during the intervention (Fig. 3; Table 2). The decrease was larger in SM than in ST during the intervention and further decreased after the intervention in the SM group. Accordingly, the mean difference (pre- vs. post-therapy) was greater in SM compared to ST, with a relative change from baseline of 34.93% versus 12.33%, respectively (Table 2). After adjustment, the mean difference of the COMFORTneo score was 3.3 points (95% CI 2.4–4.2) greater in SM than in ST.

All parameters of the COMFORTneo subscores (alertness, calmness, crying, body movement, facial tension, and muscle tone) decreased during both SM and ST interventions (Table S1). When comparing the scores at the timepoint during the intervention to the timepoint after the intervention, SM was associated with a further reduction in all six subscores, whereas ST resulted in reductions in only two of the six subscores. Nevertheless, post-therapy values for all subscores in both the SM and ST group remained lower than their respective pre-therapy values.

## Discussion

This study explored how the two interventions, live-performed sansula music as a therapeutic intervention and storytelling, affected preterm infants’ vital signs and behavioral state. Both interventions had beneficial outcomes, reflected by a decrease in heart rate, respiratory rate, and COMFORTneo score, and an increase in oxygen saturation, with stronger effects observed in infants receiving sansula music compared to those receiving storytelling.

Our findings on improved vital signs in live-performed sansula music interventions are consistent with previous research highlighting the beneficial potential of music therapy in neonatal care. However, there is a considerable heterogeneity in study designs and in how music interventions are delivered. In a study by Loewy et al. (2013)^22^, three types of live-performed music interventions were compared in preterm infants: singing a lullaby, and using the ocean disc and the gato box. Overall, positive effects on vital signs, activity, and sleep patterns were observed with slight differences among the three groups. Our findings on vital signs and behavioral state align with previous studies conducted in the same clinical setting, which used comparable methodologies, including the same musical instrument^18,20,23^. It should be noted, however, that in these earlier studies, the sansula was used in combination with improvised singing and humming. In order to apply a more standardized and universally replicable method, which is not dependent on individual vocal characteristics, we used only the instrument sansula for this study. A meta-analysis by Yue et al. (2021)^16^ analyzed 13 RCTs with a total of 1,093 preterm infants. It demonstrated significant positive effects of music interventions on heart rate, respiratory rate, stress levels, oral feeding volume, and maternal anxiety, which corresponds to our findings. In a systematic review of 39 RCTs, Costa et al. (2022)^17^ distinguished between music therapy, delivered live by a trained music therapist, and music medicine, which involves listening to prerecorded music. Both forms of music interventions showed positive effects on several outcomes in preterm infants. However, music therapy showed more consistent beneficial effects compared to music medicine in terms of cardiac and respiratory function, sleep patterns, weight gain, feeding behaviors, and brain activity. Based on these findings, Costa et al. recommend involving trained music therapists in both clinical care and research settings.

However, the findings across studies are not entirely consistent. A systematic review by Haslbeck et al. (2023)^24^ found no significant effects on respiratory rate or oxygen saturation. Nevertheless, it reported a beneficial reduction in heart rate and found no evidence of harm. The authors also highlighted uncertainty regarding the effects of music interventions on long-term development and parental anxiety.

A study investigating the potential effects on neurological outcomes in preterm infants suggest that live-performed creative music therapy, incorporating singing, humming and the vibroacoustic instrument monochord, may have positive effects on functional brain connectivity^25^. A post-hoc analysis of a study that applied a similar methodology, using singing and humming together with the instrument sansula, indicates enhanced white matter integrity in preterm infants^26^.

Our study shows that storytelling can serve as an alternative care intervention for preterm infants, but the conduction of live-performed sansula music as a therapeutic intervention was more effective. Both interventions appeared to have a calming effect on the infants, as indicated by the reduction in heart and respiratory rate and the decrease in COMFORTneo score, which includes subscores such as agitation, crying and body movement. These short-term improvements are clinically relevant, as discomfort and stress in preterm infants have been linked to long-term adverse developmental outcomes^21–23^. Although our study focused on immediate effects and did not assess long-term impact of music therapy, the calming effect and reduction of acute distress could potentially provide protective long-term advantages. However, long-term studies would be required in this field. There are several limitations to be considered when interpreting the study results. Vital signs were used to evaluate the interventions’ effects. However, these parameters are unstable in preterm infants and naturally fluctuate due to various factors like medical procedures, feeding schedules, or circadian rhythms. In our study, the interventions were not performed at a specific time of day or at specific intervals relative to other medical interventions or care activities. This could have introduced variability and potential bias. Furthermore, the use of the COMFORTneo scale, which relied solely on evaluations by the same person that was also providing the interventions, introduced a degree of subjectivity and potential observer bias. The assessment of the COMFORTneo scale was not blinded and interrater reliability was not evaluated.

Additionally, it should be noted that using the instrument sansula, rather than an intervention that includes singing, may have introduced variability in auditory input, particularly regarding the timbral and rhythmic characteristics of instrumental music versus human voice. The sansula sound may also include a vibrotactile component, representing a different type of sensory input compared with storytelling. These differences may have influenced the infants’ responses. Another limitation is that decibel levels, as well as the sound and vibration exposure near the infants’ heads, were not measured.

This study provides further evidence supporting the broader implementation of live-performed music therapy in clinical care. However, our findings also suggest that in resource-limited settings where professional therapists are unavailable, alternative caregiving approaches, such as storytelling, may provide meaningful benefits for preterm infants.

## Conclusion

This study demonstrated that both interventions, live-performed sansula music as a therapeutic intervention and storytelling, were associated with improvements in preterm infants’ physiological and behavioral parameters. Beneficial effects of SM were significantly higher than those of ST, since all analyzed outcomes (heart rate, respiratory rate, oxygen saturation, and COMFORTneo score) were positively impacted by SM. These results align with previous research and provide further evidence of the beneficial effects of live-performed music therapy on preterm infants’ health. Based on these findings, we highlight the importance of providing dedicated care and attention to preterm infants and suggest live-performed music therapy as part of NICU care.

## Supplementary Information

Below is the link to the electronic supplementary material.

Supplementary Material 1

Supplementary Material 2

## Data Availability

All data generated or analyzed during this study are included in this article and its supplementary material files. Further enquiries can be directed to the corresponding author.
