# Supplementary material for: Effects of live-performed sansula music versus storytelling on physiological and behavioral parameters in preterm infants: a randomized controlled trial
Source: Sci Rep. 2025 Nov 7;15:38995. doi: 10.1038/s41598-025-25875-8 (PMC12594991; doi:10.1038/s41598-025-25875-8)
Supplement: Supplementary file 1 — Supplementary Material 1 [file 41598_2025_25875_MOESM1_ESM.pdf]

Table S1. COMFORTneo subscores before, during, and after sansula music sessions and storytelling sessions.

**Subscore**	**Therapy**	**Sessions (n)**	**Mean before therapy (95% CI)**	**Mean during therapy (95% CI)**	**Mean after therapy (95% CI)**
Alertness	Sansula music	303	2.5 (2.4–2.6)	1.7 (1.6–1.8)	1.3 (1.2–1.4)
	Storytelling	249	2.6 (2.4–2.7)	2.1 (2.0–2.2)	1.9 (1.8–2.1)
Calmness	Sansula music	303	2.2 (2.1–2.3)	1.5 (1.4–1.5)	1.2 (1.2–1.3)
	Storytelling	249	2.3 (2.0–2.4)	1.9 (1.8–2.0)	1.8 (1.7–1.9)
Crying	Sansula music	303	1.4 (1.3–1.4)	1.1 (1.0–1.1)	1.0 (1.0–1.1)
	Storytelling	249	1.5 (1.4–1.6)	1. (1.1–1.2)	1.1 (1.1–1.2)
Body Movement	Sansula music	303	2.2 (2.1–2.3)	1.5 (1.4–1.6)	1.2 (1.1–1.2)
	Storytelling	249	2.1 (2.0–2.2)	1.9 (1.8–2.0)	1.9 (1.8–2.0)
Facial tension	Sansula music	303	2.3 (2.2–2.4)	1.6 (1.5–1.6)	1.3 (1.3–1.4)
	Storytelling	249	2.2 (2.2–2.3)	1.9 (1.8–2.0)	2.0 (1.9–2.1)
Muscle Tone	Sansula music	303	2.2 (2.1–2.3)	1.4 (1.3–1.5)	1.2 (1.2–1.3)
	Storytelling	249	2.2 (2.1–2.3)	1.8 (1.7–1.9)	1.9 (1.8–2.0)

CI = confidence interval.
