# Supplementary figures and images for: Effects of live-performed sansula music versus storytelling on physiological and behavioral parameters in preterm infants: a randomized controlled trial
Source: Sci Rep. 2025 Nov 7;15:38995. doi: 10.1038/s41598-025-25875-8 (PMC12594991; doi:10.1038/s41598-025-25875-8)

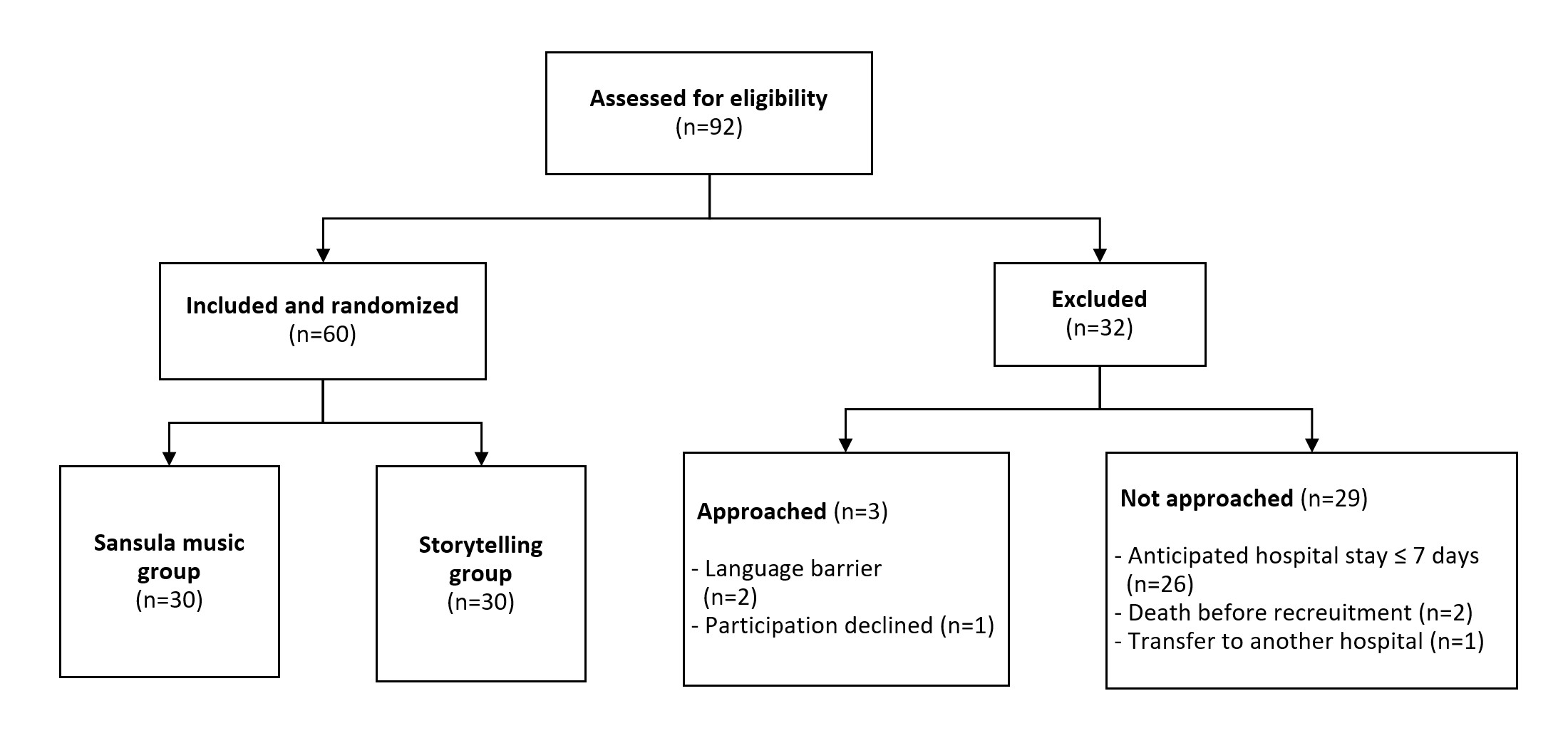

Supplement: Supplementary file 2 — Supplementary Material 2 [file 41598_2025_25875_MOESM2_ESM.jpg]
